# Charting the Spread of *Salmonella* Infection

**DOI:** 10.1371/journal.pbio.0040378

**Published:** 2006-10-17

**Authors:** Liza Gross

Every summer, local newspapers warn readers not to eat unchilled potato salad, seared hamburgers, and other picnic fare likely to precipitate an unpleasant encounter with *Salmonella enterica* bacteria. Yet in recent years, the number and severity of *S. enterica* cases has risen along with the number of factory farms (where infection can rapidly spread among tens and hundreds of thousands of animals) and the evolution of multi-drug–resistant *Salmonella* strains. Effective vaccine development and drug therapies depend on understanding how these pathogens behave inside the cell, but technical difficulties have limited scientists’ efforts to directly observe the dynamics of infection in living tissue.

In a new study, Sam Brown, Stephen Cornell, Pietro Mastroeni, and colleagues combined microscopy and dynamical modeling techniques to identify the key variables underlying infection. Their model describes pathogen proliferation at the single cell and tissue level, producing novel insights into the dynamics of infection—and providing a framework for testing antibiotics and managing antibiotic resistance.

*S. enterica* pathogens initially replicate inside phagocytic immune cells; they then escape and infect other phagocytes after bursting, or lysing, the host cell. It’s unclear what mechanisms induce lysis—programmed cell death or pathogenic poisons—or how they facilitate transmission to uninfected cells in a living organism.

In previous work, the authors imaged individual *S. enterica* bacteria within mouse liver phagocytes. They found that the number of infected cells increased along with the overall numbers of bacteria and that each infected phagocyte typically had low bacterial counts. Though bacterial growth rates differed—with virulent strains replicating faster than “attenuated” mutant strains—the bacterial distribution across cells remained near-constant, regardless of overall bacterial growth rate and time since infection. This observation raises the possibility that intracellular variations in bacterial counts result from inherent variations in phagocytes’ response to bacterial replication.

In this study, the authors used mathematical modeling to explore possible explanations for the observed distributions and spread of infection. Proliferation dynamics within and among cells was first captured in a simple model governed by two parameters: a constant bacterial division rate—so that bacterial ancestors and all descendants reproduce stochastically, with the same probability—and host cell burst size—in which the cell bursts when bacterial numbers reach a fixed value. The model assumes that when a cell bursts, each released bacteria infects a new cell. The modeling results found that many cells had just one bacterium while others had several—as they did in the mouse phagocytes—showing that heterogeneous distributions can arise from a homogenous cell population.

Still, cells could respond differently to infection, either due to intrinsic or stochastic differences among cells. The authors modeled the effects of intrinsic variations in cellular response to infection, resulting either from different replication rates or from different burst thresholds. Variable bacterial division didn’t improve the model’s fit to the phagocyte data, but variable burst threshold did, predicting that cells with virulent strains would burst at much higher bacterial densities than those with attenuated strains.

The authors then considered stochastic variations among intrinsically identical cells. Assuming that bursts occur randomly rather than at a fixed threshold, they found that reducing the rate of bacterial division reduced the rate of population growth and skewed the distribution so that host cells had fewer bacteria, like phagocytes with attenuated strains. When the authors allowed the rates of both burst and bacterial division to depend on bacterial density, the model that described their data better than all the others combined density-independent lysis with density-dependent bacterial division. This model predicts that the intracellular replication rate of *S. enterica* will decrease as the number of intracellular bacteria increases and that the bursting rate will proceed at a constant rate, with random variations, regardless of intracellular bacterial density. As before, virulent and attenuated strains behaved differently—bacterial density had a greater effect on the intracellular division rates of virulent strains.

With these insights into intra- and intercellular dynamics, the authors explored the effects of medical intervention. Since some antibiotics kill bacteria living outside cells, they refined the model to account for extracellular survival. The model showed that reducing the rate of intracellular bacterial replication leads to a reduced burst size—suggesting that the effectiveness of extracellular antibiotics would be increased if combined with antibiotics aimed at reducing intracellular replication.

These insights into the dynamic relationship between bacterial division rate, host cell lysis, and extracellular survival—and thus the probability of infecting new cells—offers a novel way of evaluating the efficacy of multi-drug therapies and the evolution of drug resistance. For example, researchers can test whether administering intracellular and extracellular antibiotics simultaneously produces synergistic effects that clear an infection. The authors hope that other researchers will incorporate new experimental results into a similar dynamical modeling approach—linking within- and among-cell microbe behavior to host response—to shed light on a wide range of host-pathogen interactions.

